# Biomineral Nano-Theranostic agent for Magnetic Resonance Image Guided, Augmented Radiofrequency Ablation of Liver Tumor

**DOI:** 10.1038/s41598-017-14976-8

**Published:** 2017-11-03

**Authors:** Anusha Ashokan, Vijay Harish Somasundaram, Genekehal Siddaramana Gowd, Ida M. Anna, Giridharan L. Malarvizhi, Badrinathan Sridharan, Rupal B. Jobanputra, Reshmi Peethambaran, A. K. K. Unni, Shantikumar Nair, Manzoor Koyakutty

**Affiliations:** 1 Center for Nanosciences and Molecular Medicine, Amrita University, Kochi, 682041 India; 2 Central Lab Animal Facility, Amrita Institute of Medical Sciences, Amrita University, Kochi, 682041 India; 3 Present Address: Department of Biotechnology, Cochin University of Science and Technology, Kochi, 682022 India

## Abstract

Theranostic nanoparticles based on biocompatible mineral compositions can significantly improve the translational potential of image guided cancer nano-therapy. Here, we report development of a single-phase calcium phosphate biomineral nanoparticle (nCP) with dual-mode magnetic resonance contrast (T1-T2) together with radiofrequency (RF) mediated thermal response suitable for image-guided RF ablation of cancer. The nanoparticles (NP) are engineered to provide dual MR contrast by an optimized doping concentration (4.1 at%) of paramagnetic ion, Fe^3+^, which also renders lossy dielectric character for nCP leading to thermal response under RF exposure. *In vivo* compatibility and dual-mode MR contrast are demonstrated in healthy rat models. MRI and T2 mapping suggest hepatobiliary clearance by ~96 hours. MRI guided intratumoral injection in subcutaneous rat glioma and orthotopic liver tumor models provide clear visualization of NP in MRI which also helps in quantifying NP distribution within tumor. Furthermore, by utilising RF mediated thermal response, NP treated tumor could be ablated using clinically approved RF ablation system (10 W,13.3 GHz). Real-time i*n vivo* thermal imaging exhibits 119 ± 10% increase in temperature change (ΔT) for NP treated orthotopic liver tumor (ΔT = 51.5 ± 2 °C), compared to untreated healthy liver control (ΔT = 21.5 ± 2 °C). In effect, we demonstrate a promising nano-biomineral theranostic agent for dual-mode MRI combined with radiofrequency ablation of solid tumors.

## Introduction

A theranostic agent is one which serves both diagnostic and therapeutic functions, a capability which will have obvious clinical advantages. With the advent of nanosystems for medical use, initial research focused separately on diagnostics and therapy. The former included quantum dots^1–3^ which faced challenges during translational phase due to toxicity concerns^4,5^ and the latter included a variety of drug delivery systems^6–11^ where drugs were incorporated into biodegradable nanoparticles for the purpose of improving therapeutic efficacy. From the last one decade, there has been interest in combining both diagnostic and therapeutic functions into a single nanosystem, referred as nanotheranostics^12–14^. It would be helpful to define two types of nanotheranostic agents. In the first type, the drug and diagnostic agent are extrinsically loaded into a nanocarrier resulting in a multi-phase nanosystem^12–15^. In the second type, the single phase nanomaterial itself has both diagnostic and therapeutic functions^16–18^. The former can be termed as ‘extrinsic’ and the latter as ‘intrinsic’ nanotheranostic systems.

Most of the reported theranostic agents are extrinsic in nature, based on polymers^19–22^, dendrimers^23–25^, liposomes^26–29^, or inorganic nanocarriers such as silica^30–32^ incorporating both diagnostic and therapeutic moieties. Limitations of such extrinsic theranostic agents include complexity of incorporating multiple materials into a single nanosystem and challenges in obtaining high encapsulation efficacy. In contrast, intrinsic systems are relatively simple, single-phase materials with multi-functionality. Superparamagnetic iron oxide nanoparticles (SPIONs) is the only known intrinsic theranostic nanosystem reported so far for combining T2 weighted (dark) MR contrast together with magnetic hyperthermia property^18,33^. Although SPIONs is approved for T2 MR imaging, its application is limited due to prolonged *in vivo* clearance time (>30 days)^34^ and iron overload related ROS generation^35,36^.

Our group has been working in the area of biomineral nanocontrast agents since 2008^37–39^, especially on calcium phosphate/apatite nanoparticles, that has great potential for clinical translation due to their inherent compatibility as calcium phosphate is the major mineral component in human bone. Our earlier work focused on the development of multiple ions doped calcium phosphate nanoparticles (nCP) showing combined optical, magnetic and nuclear contrast properties^37–39^. Barth *et al*. used calcium phosphosilicate nanoparticles loaded with indocyanine green for targeted tumor imaging^40^ and photodynamic therapy^41^ in animal tumour models. Recently, the capability of calcium phosphate to dissolve at acidic pH was utilised by Mi P *et al*. to develop pH-activatable manganese doped calcium phosphate nanoparticle that showed enhanced magnetic contrast within acidic tumor microenvironment^42^. Calcium phosphate nanoparticles were also incorporated with a fluorophore along with drug, ceramide, for development of an extrinsic theranostic agent^43^. Our present work shows the potential of nCP to provide contrast and inherent therapeutic potential as an intrinsic theranostic agent.

In the present work, we optimized a unique intrinsic nanotheranostic property for nCP doped with 4.1 at% of Fe^3+^ (nCP:Fe) showing simultaneous dual-mode T1-T2 MR contrast and RF mediated thermal response suitable for MRI assisted RF ablation therapy of cancer. Dual-mode MRI helps in combining the merits of both T1 and T2 relaxivity values of the tissue^44^. T1 mode provides excellent soft tissue contrast together with anatomical details whereas T2 mode helps in the identification of lesions. So far, either paramagnetic gadolinium complexes^45^ or SPIONs^46^ were separately used for T1 or T2 imaging, respectively. Recently there were efforts to develop dual mode nanoconstructs by complexing^47^ or doping Gd^3+^ with FeO^48^. Instead of using heavy-metals such as Gd^3+^, a potential nephrotoxic element, dual mode MR contrast using low-dose Fe^3+^ doped (1.5–4.1 at %) biomineral calcium phosphate (nCP) may be a better alternative. Most importantly, Fe^3+^ doped nCP showed unique dielectric-loss characteristics under RF exposure, resulting in increase in temperature on exposure to low-power (<10 W) radio-waves. Radiofrequency ablation (RFA) is clinically utilised for minimally invasive ablation of solid tumors including hepatocellular carcinoma (HCC) and renal cancer. A major drawback of current RFA is the inferior thermal conductance of tissue, limiting the size of the lesion that can be treated to <5 cm^49^. In clinics, this problem is addressed by repeated repositioning of RF electrodes in order to cover the entire disease area or by intratumoral injection of ionic solutions such as saline to improve RFA efficiency^50^. There are also a few reports where nanostructures such as nano-gold^51^, carbon nanotubes^52^ and graphene^53^ were utilized for enhancing the efficacy of RFA. However, all these materials are non-biodegradable and may pose long-term toxicity effects. Furthermore, there are no theranostic nanoparticles reported so far for minimally invasive, MR image guided RFA. Although, Merkle *et al*. investigated the feasibility of using FeO nanoparticles for MR guided RFA of liver, there was no significant increase in coagulation diameter observed *in vivo* ^33^. To the best of our knowledge, this is the first report on single-phase bio-mineral intrinsic theranostic nanoparticle providing dual mode MR contrast and RFA response.

## Results and Discussion

### Synthesis and Characterization of nCP:Fe

Our main objective was to optimize a therapeutic functionality in Fe doped nCP together with dual mode T1 and T2 MR contrast property. In our previous reports on doped nCP for multimodal imaging^37–39^, we used Gadolinium as the doping agent for MR contrast. In the present work, instead of Gd^3+^, which may cause nephrogenic systemic fibrosis in patients with chronic kidney disease^54^, we used a more biocompatible Fe^3+^ to generate paramagnetic property in nCP. We hypothesize that at appropriate doping concentrations, Fe^3+^ can also induce lossy dielectric character for nCP at radio-frequency range that would make the material suitable for RFA.

**Figure 1:**
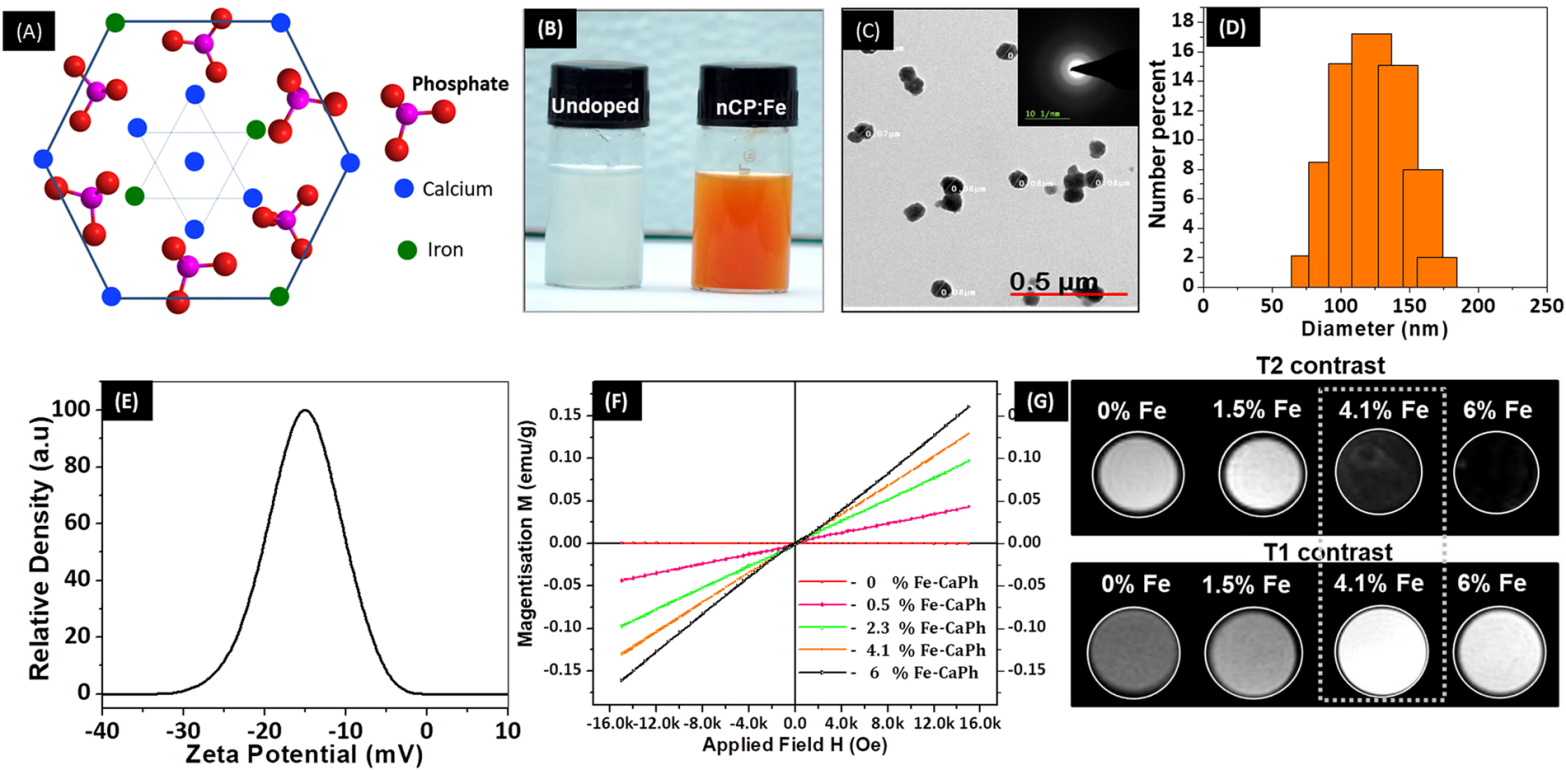
Characterization of nCP:Fe. (**A**) Schematic of nCP:Fe where a few Ca^2+^ atoms are replaced by Fe^3+^ (**B**) Photograph of undoped nCP (left) and nCP:Fe (right) solution at concentration of 10 mg/mL. (**C**) TEM image of nCP:Fe, Inset: ED pattern (**D**) DLS showing size distribution (**E**) Zeta potential data (**F**) VSM data of different batches of nCP:Fe doped with varying concentration of Fe^3+^. (**G**) T2 and T1 weighted MR contrast of 1.25 mg/mL of nCP:Fe doped with varying concentration of Fe^3+^, dispersed in agar phantom. 4.1% Fe doped nCP showed an optimized dual T1-T2 contrast that is marked in white dotted box.

Figure 1A shows the schematic lattice arrangement of nCP:Fe where Fe^3+^ possibly substitute the cationic position of Ca^2+^ or occupy interstitial space. An obvious change in body-color from white (undoped nCP) to brown was observed after doping with Fe^3+^ (Fig. 1B). TEM images (Fig. 1C) showed well dispersed spherical nanoparticles of average size ~90 nm which was confirmed from DLS analysis (Fig. 1D) that gave a size distribution of 125 ± 60 nm. X-ray diffraction (Supplementary Figure S1A) and electron diffraction pattern (Inset Fig. 1C) indicated amorphous nature of nCP:Fe. During the reaction between calcium and phosphate precursors for nCP:Fe synthesis, it was critical to maintain the pH at ~9 for obtaining spherical nanoparticles, as neutral or acidic pH (pH 5–7) resulted in formation of rod shaped particles as reported earlier by our group^38^. In order to prevent the aggregation and crystal growth after synthesis, we surface capped the nanoparticles with citrate ions. Adsorption of citrate ions on calcium phosphate facets contributes to controlled growth of nanoparticles^55^. This interaction was also reflected in the relatively high negative zeta potential, −15 mV of capped nCP:Fe (Fig. 1E). Inductively coupled plasma (ICP) analysis showed an average doping efficiency of ~40% (Table 1). Further, we also investigated the washing out of Fe^3+^ ions from nCP:Fe, for which the final supernatant obtained after fourth wash of sample was analyzed by ICP. Fe^3+^ level in supernatant was below 1 ppm, indicating no significant leakage of Fe^3+^ from nCP:Fe. Magnetic property analyzed by VSM showed linear increase in magnetization with external field, indicating paramagnetic behavior compared to the diamagnetic property of undoped nCP (Fig. 1F). With increase in doping concentration from 0.5 to 6 at% an increase in paramagnetic behavior (Fig. 1F) and magnetic susceptibility (Supplementary Figure S1B) was observed. There are reports on the paramagnetic^56^ or superparamagnetic^57^ behavior of Fe^2+^ or Fe^3+^ incorporated hydroxyapatite depending on the ionic state of doped Fe and phase of the host matrix. The paramagnetic nature of nCP:Fe in the present case may be conferred to the presence of lone unpaired electron in the 3d orbital of Fe^3+^.

**Table 1:** Actual Fe^3+^ doping % (calculated from ICP data) compared to % Fe^3+^ added during synthesis.

% Fe^3+^ added during synthesis	% Fe^3+^ doped within nCP (ICP data)
1	0.5
5	2.3
10	4.1
15	6

### Dual mode T1-T2 weighted MRI of nCP:Fe

Next, we investigated the T1 and T2 weighted magnetic contrast property of nCP:Fe. Contrast property of paramagnetic nanoparticles is highly sensitive to the concentration of doped ions^58^. The T1 effect (bright contrast) dominates at lower concentrations of paramagnetic impurity due to spin-lattice interactions between the paramagnetic ions and protons whereas T2 effect (dark contrast) dominates at higher concentrations due to spin-spin interactions between high concentration of paramagnetic ions^37^. Utilizing this property of paramagnetic ions, in the present work, we optimized Fe^3+^ doping concentration to obtain an enhanced T1 and T2 relaxation rates that would lead to both bright contrast in T1 mode and dark contrast in the T2 mode of MRI. Figure 1G shows that an increase in Fe^3+^ dopant concentration from 1.5 to 6 at% resulted in an enhancement of T2 contrast intensity whereas T1 contrast was maximum at 4.1 at% which reduced with higher doping. This suggests that spin-lattice relaxation rate dominated till 4.1 at% doping after which spin-spin relaxation rate was higher leading to T2 contrast. Therefore we optimized the doping concentration at 4.1 at% to obtain an effective T1 and T2 relaxation rates suitable for dual mode T1-T2 contrast in MRI. Mapping studies carried out to estimate the T1 and T2 relaxivity values of 4.1 at% Fe doped nCP gave *r1* = 0.75 mM^−1^s^−1^ and *r2* = 29.6 mM^−1^s^−1^ (Supplementary Figure S1C, D). Although these values are relatively lower than that of typical contrast agents (Gd-DOTA: *r1* = 2.8 mM^−1^s^−1^, SPIO: *r2* = 213 mM^−1^s^−1^ at 7 T), this is optimum for the development of a dual mode T1-T2 contrast agent for magnetic contrast assisted intratumoral injection for MR guided therapeutics in clinics. Thus, we selected 4.1 at% Fe^3+^ doped nCP as optimum for dual mode T1-T2 contrast imaging in MRI and this sample is hereafter referred as nCP:Fe.

### *In vitro* compatibity of nCP:Fe

Prior to *in vivo* studies, we tested the compatibility of nCP:Fe towards primary human peripheral blood derived mononuclear cells (PBMC) and RBC. Blood compatibility assessment is a prerequisite for preclinical testing of nanoparticle derived medicines and medical devices. PBMC viability analysis showed excellent compatibility up to 500 µg/mL after 48 hours of treatment (Supplementary Figure S2A). Hemolysis assay (Supplementary Figure S2B) also showed that nCP:Fe does not disturb the RBC membrane integrity up to a tested concentration of 250 µg/mL which was further confirmed by SEM imaging (Supplementary Figure S2B Inset). It was also important to check the ROS generation capability of nCP:Fe as there are a few reports that discuss the possible stress mechanism activated by SPIONs which is ROS mediated^36^. DCFH-DA mediated ROS analysis in N1S1 cells showed that nCP:Fe did not induce any additional ROS (11.3 ± 1.5%) compared to PBS control (12.5 ± 0.5%) (Supplementary Figure S2C, D) whereas ~100% of hydrogen peroxide treated cells produced significant ROS (Supplementary Figure S2E). The compatibility of nCP:Fe to the blood cells and low ROS generation may be positively considered for using nCP:Fe as an alternate MR contrast agent.

### *In vivo* dual mode (T1-T2) MR Imaging in healthy rat and tumor models

**Figure 2:**
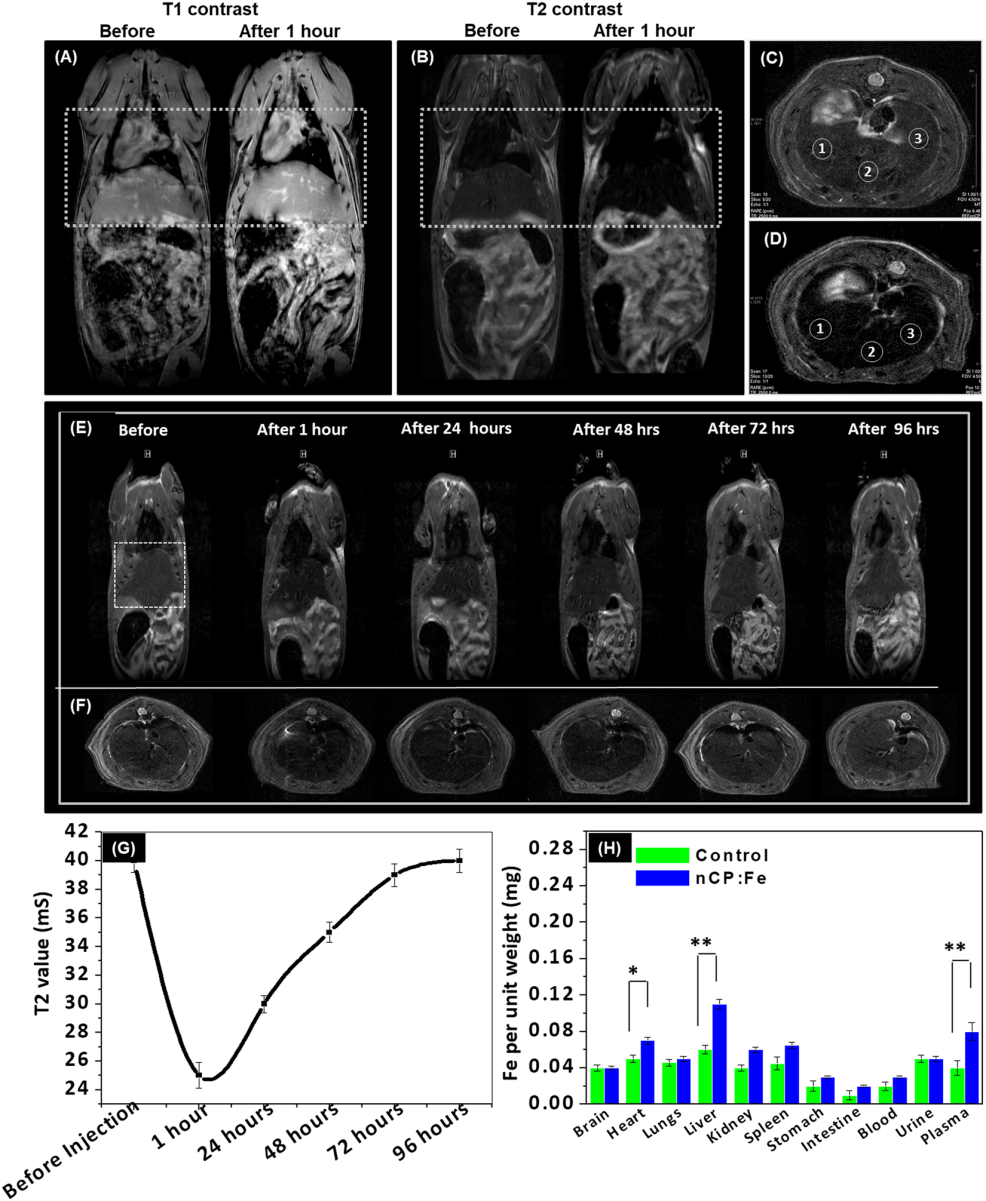
*In vivo* dual T1-T2 contrast and biodistribution of nCP:Fe. *In vivo* (**A**) T1 weighted and (**B**) T2 weighted MRI (coronal section) of Wistar rat before and after nCP:Fe injection. Enhancement of both T1 and T2 contrast can be observed after sample injection in liver and heart region (white dotted box) (**C**) Axial liver section before sample injection (**D**) Axial liver section 30 minutes after sample injection (**E**) T2 weighted MRI of wistar rat over a period of 96 hours after intravenous injection of nCP:Fe (**F**) Corresponding axial liver sections over a period of 96 hours (**G**) Variation in T2 time of liver over a period of 96 hours after nCP:Fe injection (**H**) Fe content in different organs, 1 hour after sample injection, compared to PBS control, estimated by ICP analysis.

Reports on *in vivo* dual mode MRI used either two separate contrast agents injected one after the other^59^ or an extrinsic nanoparticle incorporating two different magnetic impurities (Gd and SPIO) for T1 and T2 contrast^47,48^. To evaluate the *in vivo* dual mode T1-T2 contrast property of nCP:Fe, 10 mg/kg sample was intravenously injected to healthy rats. After 30 minutes, an enhancement of both T1 and T2 contrast was observed especially in the liver and heart region as shown in white dotted box in Fig. 2A,B. T2 mapping analysis showed that reduction in T2 contrast in liver region was also associated with lowering of T2 relaxation time from 40 ± 1 mS to 25 ± 1 mS. Axial T2 weighted images of liver sections before (Fig. 2C) and after (Fig. 2D) nCP:Fe injection clearly showed enhancement in T2 contrast intensity. T2 mapping data (Supplementary Figure S3) obtained from selected ROI (white circles in Fig. 2C,D) reflected the enhanced relaxivity changes in the liver after nCP:Fe injection.

We also evaluated the biodistribution and clearance time of nCP:Fe after intravenous injection. T2 weighted whole body coronal (Fig. 2E) and axial (Fig. 2F) MRI showed an increase in T2 contrast in liver (shown in white dotted box) within the first one hour which gradually reduced to initial (before injection) contrast by 96 hours. This variation was also reflected in T2 relaxation values that drastically reduced in the first hour after injection (from 40 ± 1 mS to 25 ± 1 mS) and then gradually increased to initial value 40 mS by ~96 hours (Fig. 2G). This clearly indicates initial nanoparticle uptake by mononuclear phagocyte system within the liver and subsequent clearance over a period of 96 hours. This was confirmed by tissue-ICP analysis after 1 hour of injection which showed an increase in Fe^3+^ content in all organs as well as plasma with significantly higher concentration in the heart, plasma and liver (Fig. 2H). We believe that the increase in Fe content in plasma is not due to the degraded nanoparticles because our *in vitro* studies on nCP:Fe treated RAW 264.7 macrophages showed less degradation even after 12 hours of intracellular localization (Supplementary Figure S4). These results suggest that the nanoparticles circulated in the body and accumulated in the liver within 1 hour and slowly cleared by 96 hrs. We compared the clearance time of nCP:Fe vs SPIONS (in healthy rats) after intravenous injection using T2 values of liver and found that nCP:Fe cleared within 4–5 days whereas SPIONS was not cleared upto 15 days (Supplementary Figure S5). Our previous observation on ICG and Gd^3+^ co-doped calcium phosphate nanoparticles indicated hepatic clearance of nCP^37^. Compared to the changes observed in the T2 relaxation values of liver, post nCP:Fe injection, we have not observed any significant contrast changes in kidneys. Therefore we assume that the nCP:Fe nanoparticles were cleared through the hepatobiliary route similar to ICG and Gd doped nCP.

**Figure 3:**
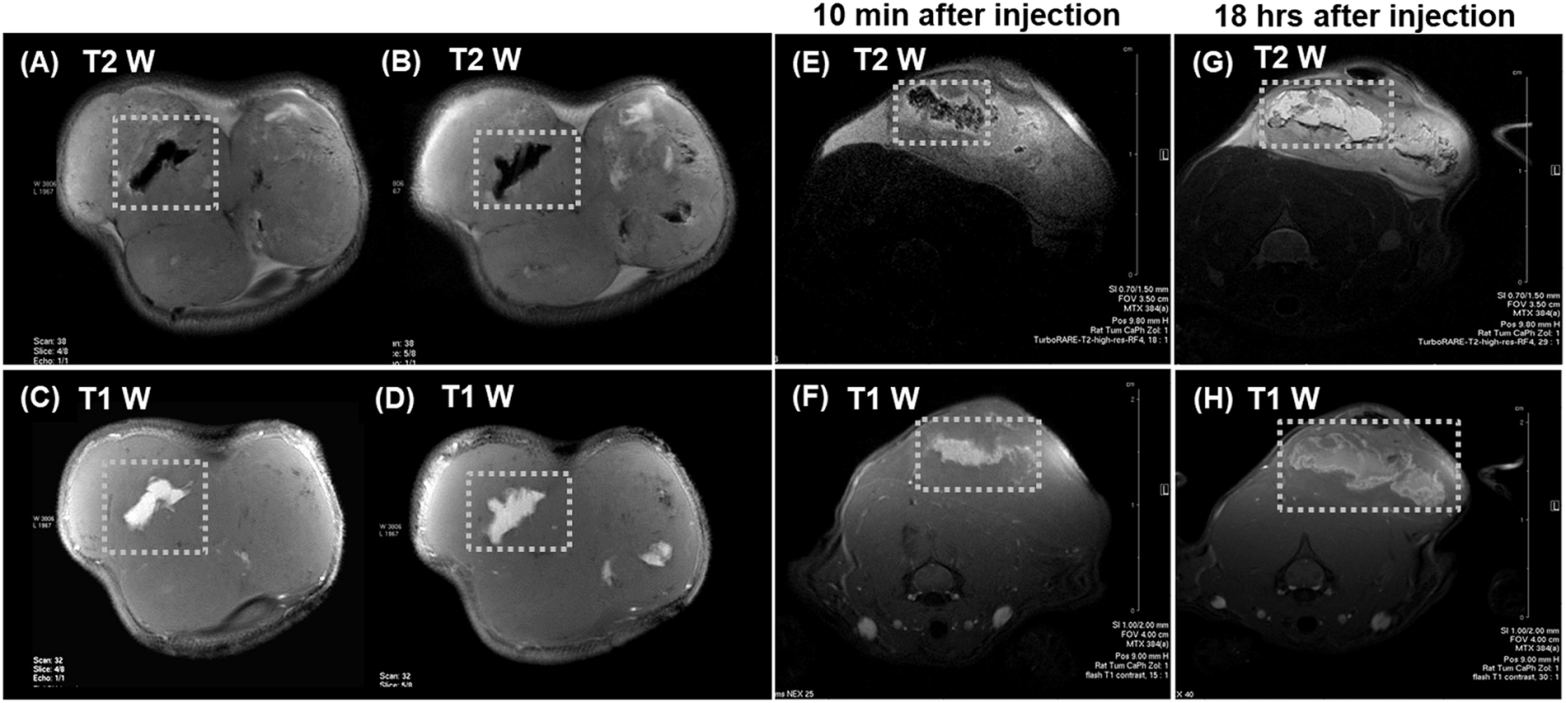
Dual T1-T2 contrast variation after intra-tumoral injection. (**A**,**B**) T2 weighted and (**C**,**D**) T1 weighted MR image (coronal section) of subcutaneous tumor after intratumoral nCP:Fe injection. (**E**) T2 weighted and (**F**) T1 weighted MRI of subcutaneous tumor (axial sections) 10 mins after intratumoral injection of nCP:Fe. (**G**) T2 weighted and (**H**) T1 weighted MRI of subcutaneous tumor (axial sections) 18 hours after intratumoral injection of nCP:Fe. Sample injected regions are shown in white dotted boxes.

Considering the potential clinical application of intratumoral nCP:Fe injection for MRI assisted radiofrequency ablation therapy, we tested its ability to provide dual mode contrast in the tumor region after direct injection into the subcutaneous tumor mass. 10 mg/kg of sample was injected to the tumor, grown in Wistar rat model. Excellent T2 (Fig. 3A,B) and T1 contrast (Fig. 3C,D) was observed from the sample injected region as shown in two different slices. The contrast change also correlated with the reduction of relaxation times, T2 from 48 ± 2 to 18 ± 1 mS and T1 from 2096 ± 3 to 340 ± 2 mS. Interestingly, imaging after 18 hours showed an increase in distribution of nanoparticles from 0.046 ± 0.001 cm^3^, 10 minutes after injection (Fig. 3E,F), to 0.056 ± 0.005 cm^3^, 18 hours after injection (Fig. 3G,H). This also led to changes in contrast properties. Dark contrast in T2 mode (Fig. 3E) turned bright (Fig. 3G), clearly indicating dilution of paramagnetic nanoparticles due to the increased diffusion into larger tumor area. This was also noted in T1 imaging (Fig. 3F,H) and mapping, as T1 relaxation time increased from 340 ± 2 mS (at the time of injection) to 840 ± 4 mS (after 18 hours). By normalizing the tissue contribution to T1 value and approximating the tumor as a homogenous tissue, we have calculated the concentration of nanoparticles distributed over the tumor region, from the T1/T2 values compared to the injected dose (3 mg). We found that ~2.5 mg was the distributed dose after 18 hours in a tumor volume of 0.056 ± 0.005 cm^3^. Thus, we demonstrated excellent T1 and T2 contrast property of nCP:Fe injected intratumoral regions and showed its capability to diffuse throughout the tumor region up to 1–1.8 cm, which could be quantified by T1/T2 mapping. The capacity of nanoparticles to distribute throughout tumor and the ability to view and quantify the dose present at tumor site in a specified time is critical in implementing theranostic dosimetry.

### *In vitro* radiofrequency response of nCP:Fe

**Figure 4:**
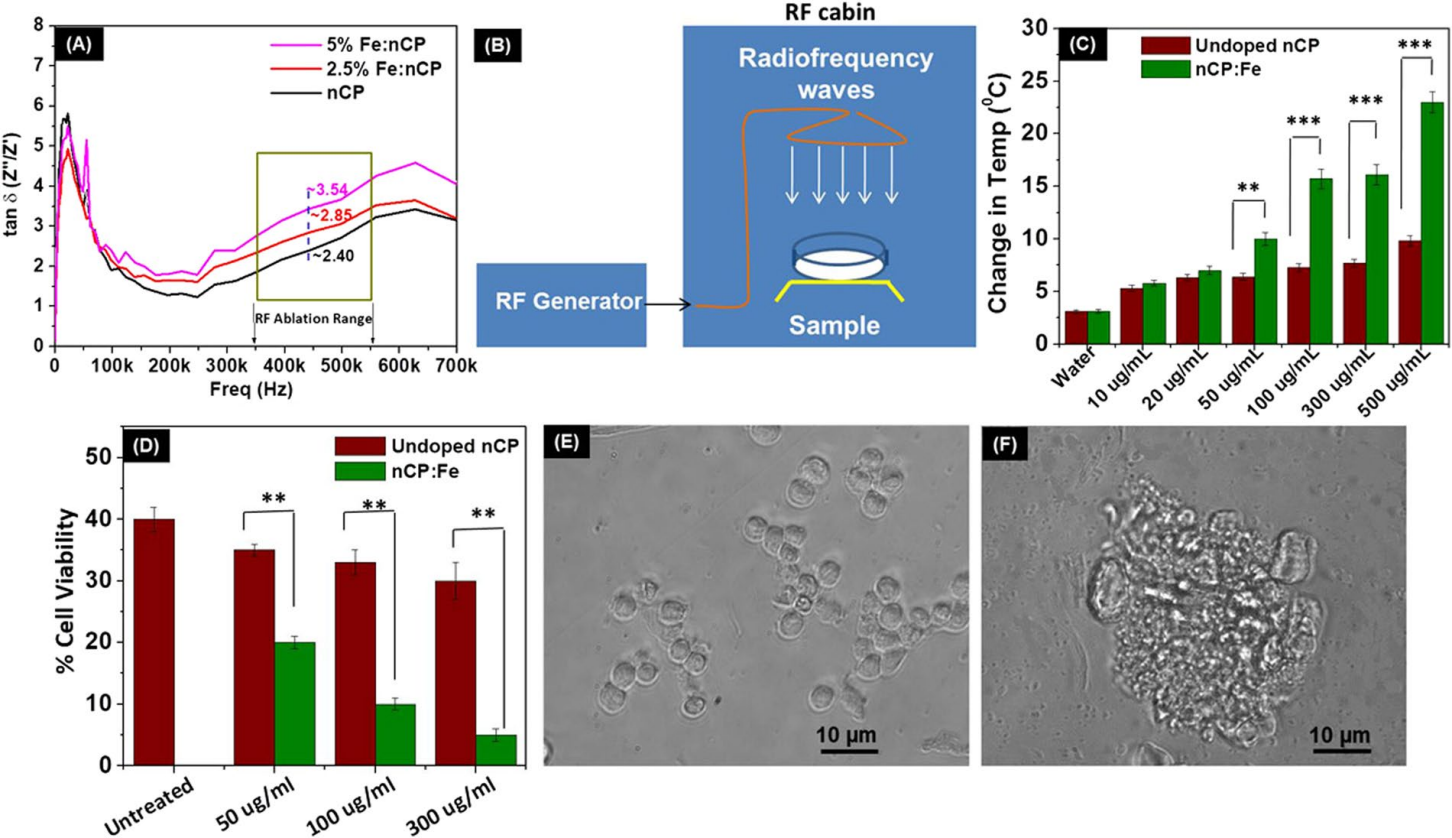
Radiofrequency response of nCP:Fe and its effect on cell viability. (**A**) Impedance spectroscopy analysis showing variation of tan δ value of nCP:Fe with frequency (**B**) Schematic of non-invasive RF system (**C**) Change in temperature obtained by RF exposure of different concentration of nanoparticles at 100 W for 1 minute. (**D**) Viability of nCP:Fe/nCP treated N1S1 hepatoma cells, 4 hours after RF treatment (100 W power was applied for 5 minutes). Viability test was done 48 hours after RF exposure. Optical microscope images of (**E**) untreated (**F**) nCP:Fe treated cells, 48 hours after RF exposure.

After the optimization of MR contrast properties and tissue distribution, we investigated if the doping of Fe within nCP changed its dielectric loss property which could be utilized for its application in RFA. Variation in dielectric loss was tested using impedance analyzer for a frequency range of 0–700 kHz. We found that in the frequency range applied for RF ablation (350–550 kHz), the dielectric loss, tan δ, increased from 2.4 (undoped nCP) to 3.54 (4.1% Fe doped nCP) at 450 KHz (Fig. 4A). This clearly indicated enhanced dielectric loss character due to Fe^3+^ doping in the bioceramic, nCP. Earlier reports on the dielectric loss properties of calcium phosphosilicate glass doped with iron oxide (Fe_2_O_3_) was attributed to the increased ionic conductance of iron in a deformed calcium phosphosilicate structure^60^. We expect similar features in nCP:Fe, where doped Fe may have increased the ionic conductance of nCP:Fe leading to increase in dielectric loss. Further, we compared the RF mediated heating of undoped nCP and nCP:Fe at varying NP concentration at 100 W RF for 1 minute exposure using a non-invasive RF machine (Schematic: Fig. 4B). In the concentration range of 50–500 µg/mL, significant increase in solution temperature leading to temperature rise, ΔT, 22 ± 1.5 °C (maximum temperature 50 °C) was observed for nCP:Fe compared to ΔT < 10 °C (maximum temperature 38 °C) for undoped nCP (Fig. 4C). Rise in temperature up to 50 °C will be sufficient to initiate an ablative response in tumor cells^61^.

**Figure 5:**
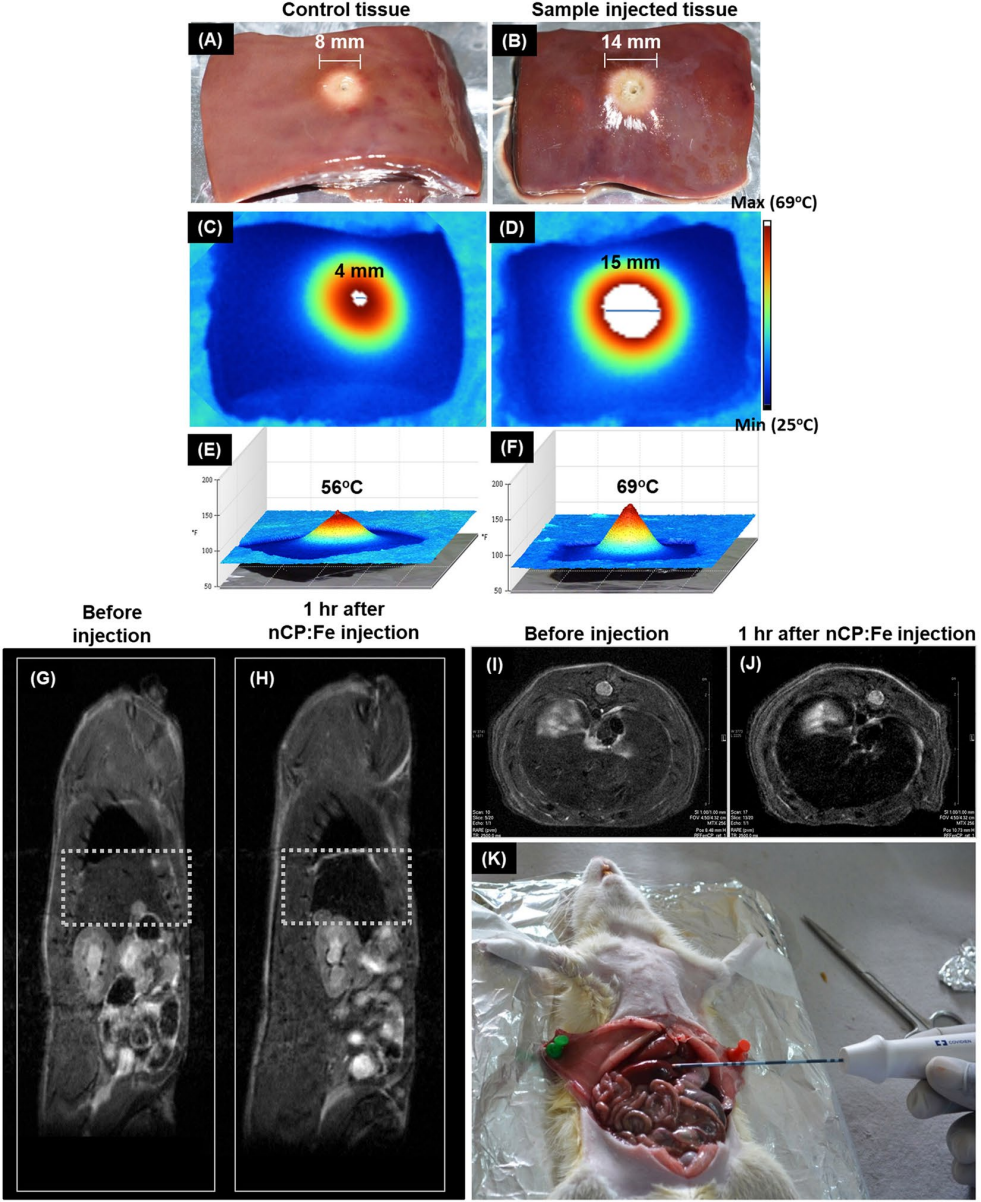
Radiofrequency ablation of phantom goat liver and healthy rat liver tissue after nCP:Fe injection. (**A**) Control (PBS injected) goat liver phantom tissue after RFA (ablation diameter 8 ± 1 mm) (**B**) nCP:Fe injected phantom tissue after RFA showing enhanced ablation diameter of 14 ± 2 mm (**C**–**F**) Corresponding IR images and 3D temperature plots of the ablated tissue (**C**,**E**) Control tissue after RFA gave peak temperature of 56 ± 2 °C, hot-spot diameter of 4 ± 1 mm (**D**,**F**) Sample injected tissue after RFA gave peak temperature of 69 ± 2.5 °C, hot-spot diameter of 15 ± 1 mm. T2 weighted MRI (coronal section) of rat (**G**) before and (**H**) 1 hour after nCP:Fe injection. Liver is shown in dotted box. T2 weighted MRI of liver (axial section) (**I**) before and (**J**) 1 hour after sample injection (**K**) RF ablation of rat liver being carried out using clinically used cooled tip RF probe.

To evaluate RF mediated cell death under *in vitro* conditions, N1S1 hepatoma cells were treated with both nCP and nCP:Fe for 4 hours followed by non-invasive RF treatment (100 W, 5 minutes exposure). Figure 4D shows significant reduction in viability for nCP:Fe treated cells compared to that of undoped nCP. 300 µg/mL of nCP:Fe reduced the cell viability to 5 ± 0.5% whereas 31 ± 2% of cells remained live when treated with same concentration of undoped nCP. Optical images of control cells showed round morphology 48 hours after RF treatment (Fig. 4E) whereas nCP:Fe treated cells lost membrane integrity and was found disintegrated (Fig. 4F). Next, we investigated the RF response in goat liver phantom tissue. Goat liver was collected from 3 different animals and divided into two groups (nCP:Fe group and PBS control group) each containing 2 phantoms from each animal. We injected samples at a concentration of 10 mg/kg to the tissue at a fixed depth of 1 cm and applied RF (10 W for 1 minute) using clinical RFA system. 87% increase (8 ± 1 mm for PBS control group to 14 ± 2 mm for nCP:Fe group) in the diameter of RF ablated necrotic area was noted for nCP:Fe injected tissue phantom (Fig. 5B) compared to the PBS injected control tissue (Fig. 5A). This temperature difference between the ablated tissues was imaged in real-time using a hand-held IR thermal imaging camera. The images (Fig. 5D,F) clearly showed that nCP:Fe injected tissue heated to a peak temperature of 69 ± 2.5 °C whereas the control tissue was heated to only 56 ± 2 °C (Fig. 5C,E). The hot-spot (maximum temperature region) diameter for nCP:Fe was 15 ± 1 mm (Fig. 5D) versus 4 ± 1 mm (Fig. 5C) for PBS control. On varying the angle between the sample and IR camera, there is a possibility for change in temperature recorded at different angles. Therefore we calculated the error that can happen by measuring a constant temperature (dish of distilled water heated to 35 °C) at different angles (0° to 90°) and found that the maximum deviation is ± 0.45 °C (Supplementary Figure S6). This is well within the error value reported ( ± 1.5 °C) in the mean temperature value in IR images. Here we used a plane surface (dish of distilled water) to calculate error, whereas in *in vivo* conditions the surfaces are curved that can increase the error value. Therefore, whenever possible, the angle of the IR camera should be maintained perpendicular to the curved surface under analysis. Thus the phantom tissue data clearly showed enhanced ablative effects of nCP:Fe compared to PBS. This ablative effect combined with dual mode (T1-T2) MR contrast properties reveals the potential of using nCP:Fe for MRI guided RFA.

### *In vivo* MRI assisted RFA of healthy liver and orthotopic liver tumor

**Figure 6:**
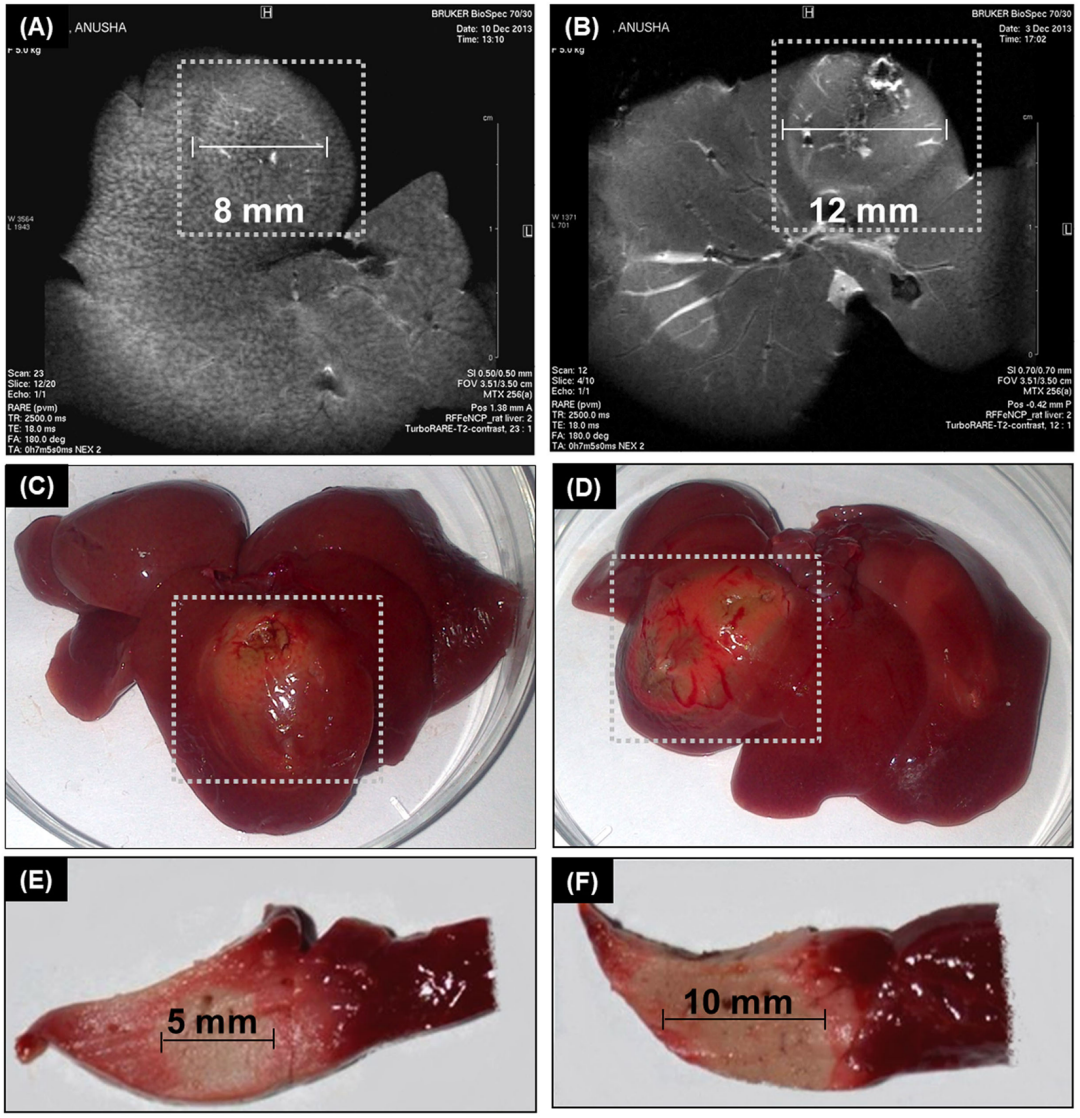
Response to RFA evaluated by MRI and viability staining. T2 weighted MRI of liver (axial section) after RF exposure (**A**) liver from control animal injected with PBS (**B**) liver from animal injected with nCP:Fe. Region of necrosis is shown in white dotted box (**C**) Photograph of liver from control animal and (**D**) liver from sample injected animal. Region of necrosis is shown in white dotted box. Viability staining by TTC of liver sections from (**E**) control animal (**F**) animal injected with nCP:Fe.

To evaluate the capability of nCP:Fe to provide MR image assisted application of RFA *in vivo*, 10 mg/kg nCP:Fe was intravenously injected to Wistar rat. Two groups (PBS control group and nCP:Fe group), each containing 3 animals were taken. Sagittal whole body (Fig. 5 G,H) and axial liver MRI (Fig. 5 I,J) showed clear T2 contrast enhancement in the liver, one hour after nCP:Fe injection. RFA was then carried out under anesthesia at 10 W power for 1 minute as shown in Fig. 5K. After RFA, MRI of axial liver section showed contrast enhancement in the necrotic region for the PBS control (Fig. 6A) and nCP:Fe injected (Fig. 6B) animal. The diameter of RFA induced necrosis was measured as 8 ± 1 mm for control (Fig. 6 A,C) and 12 ± 1 mm (Fig. 6 B,D) for sample injected animal. MR images (Fig. 6B) clearly demarcated the necrosed region in nanoparticle injected animal compared to the control (Fig. 6A), indicating the enhanced T2 effect contributed by the NP. This is clinically relevant because in MR guided saline perfused RFA, the saline injection reduces MR contrast which made it difficult to assess the extent of necrosis^62^. In the present case, nCP:Fe mediated contrast enhancement helped to better evaluate the actual therapeutic response by way of measuring the extent of coagulation necrosis during and after RFA. RF mediated necrosis was also confirmed by tissue-viability staining using triphenyltetrazolium chloride (TTC) that stains viable tissue pink and leave the necrosed tissue unstained. TTC staining showed an increased area of complete necrosis (unstained area of ~10 mm) in the nCP:Fe injected liver section (Fig. 6F) compared to control (unstained area of ~5 mm) (Fig. 6E). Histological analysis of the liver tissue confirmed the RF mediated necrosis in nanoparticle treated liver (Arrow mark, Supplementary Figure S7B), which is showed in comparison to healthy liver region without RF treatment (Supplementary Figure S7A).

**Figure 7:**
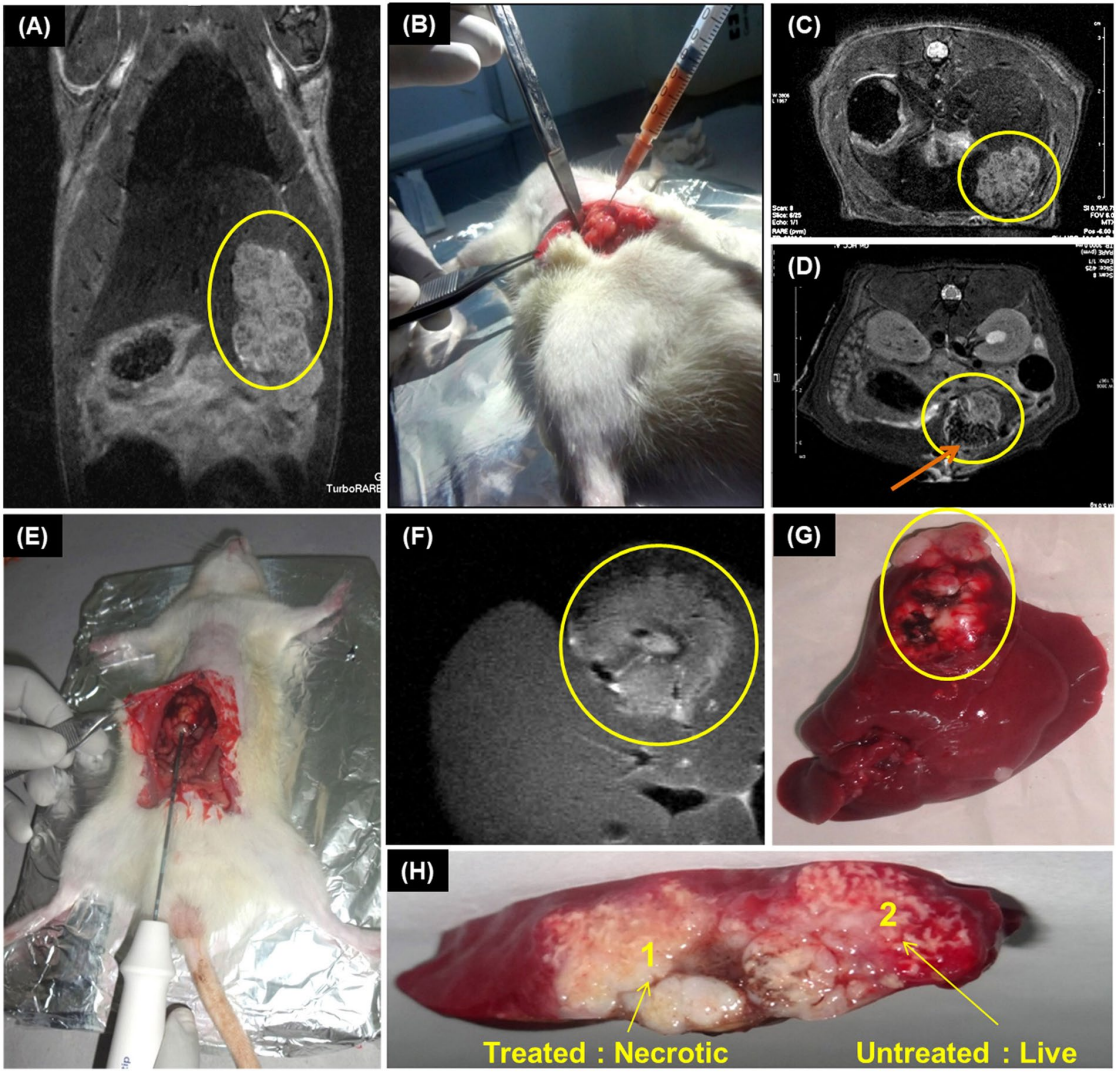
MRI assisted RFA in orthotopic liver tumor model. (**A**) Coronal T2 weighted MRI of tumor model showing liver tumor in encircled region (**B**) Intratumoral injection of 300 µL of 10 mg/ml nCP:Fe. T2 weighted axial MRI (**C**) before and (**D**) after intratumoral sample injection. Tumor region marked within yellow circle. The dark region within the tumor (arrow mark) is due to T2 contrast of nCP:Fe. (**E**) RFA of the liver tumor. Probe was inserted to tumor (at intersection of injected and non-injected area), 10 W power was applied for 1 minute (**F**) Axial MRI of the liver showing the bright ablated region (**G**) Photograph of the rat liver showing ablated tumor within circle (**H**) TTC stained liver section showing sample treated necrotic tumor (unstained: Area 1) and untreated control tumor showing viability (stained pink: Area 2).

Next, we investigated the efficacy of nCP:Fe for MR assisted RFA in orthotopic liver tumor (N1S1) model. Considering intratumoral injection of RF enhancers in clinics, we directly injected nCP:Fe into tumor lobe which was followed by MR assisted RFA. For proof of concept, we carried out the experiment in 3 orthotopic rat liver tumor models. The tumor was well demarcated in the coronal whole body (Fig. 7A) and axial liver (Fig. 7C) MRI and marked in yellow circles. In order to keep a control region within the same tumor, we have considered two different tumor lobes of same tumor for the study. nCP:Fe was injected to the lower tumor lobe (Fig. 7B) resulting in excellent dark contrast in T2 weighted axial liver MRI section (arrow in Fig. 7D). RFA was applied at the interface of sample injected and non-injected lobe (Fig. 7E) using a clinical RF probe at a low dose of 10 W for 1 minute. The necrosis due to ablation was clearly seen in the MRI (Fig. 7F). Treated liver was resected out (Fig. 7G) and viability staining by TTC was done to examine the extent of ablation. Figure 7H clearly showed specific areas of necrosis (no stain area, Mark-1) in nCP:Fe injected tumor lobe compared to the untreated control lobe (pink stain area, Mark-2). This experiment also showed that nanoparticles remained well within the injected region of tumor and effect of RFA was confined to the same region. This justifies our proposal to administer nCP:Fe as intra-tumoral formulation rather than intravenous injection where healthy liver cells may also take up nanoparticles and undergo non-specific ablation.

**Figure 8:**
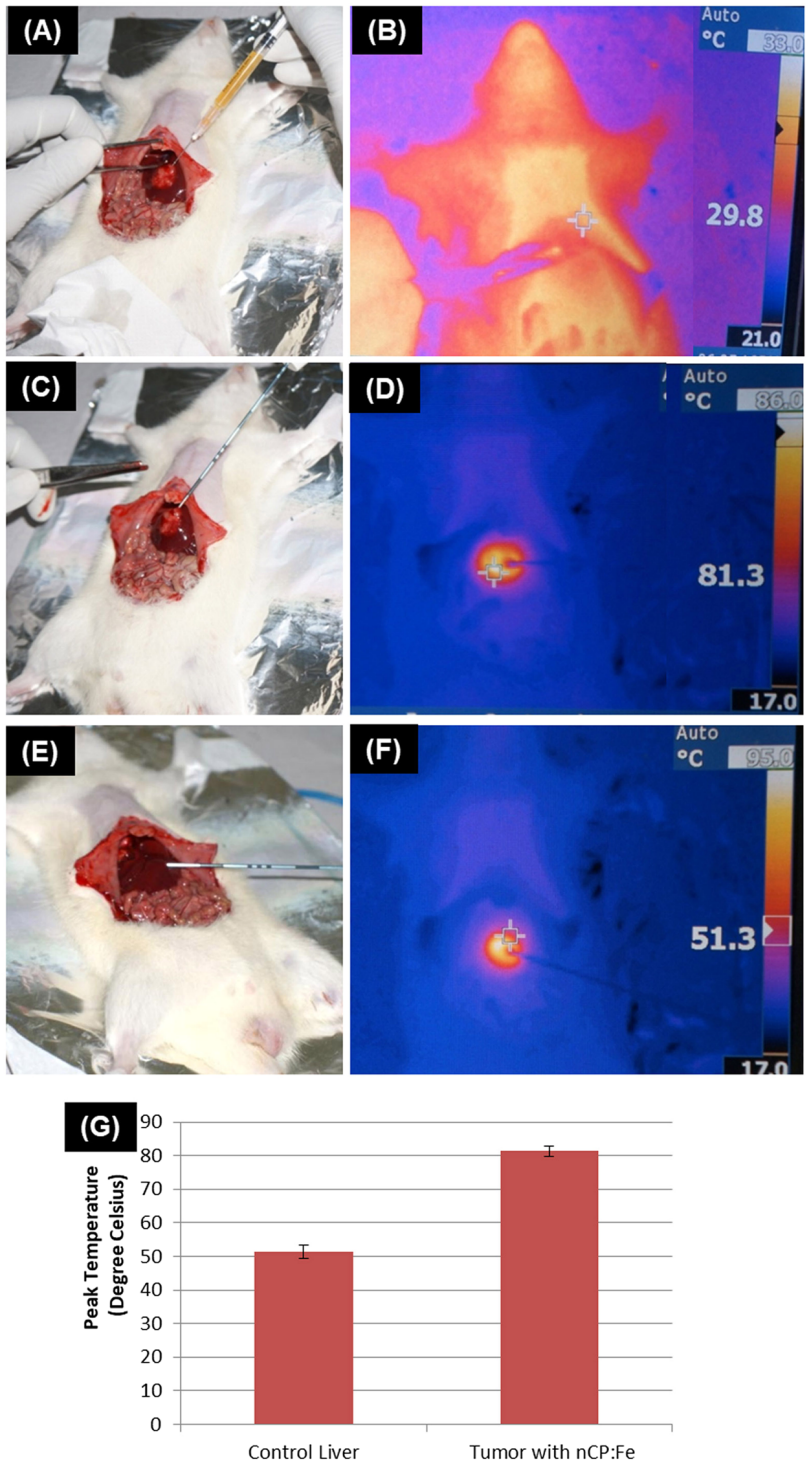
RFA of orthotopic liver tumor model and corresponding infrared images in comparison with healthy liver. (**A**) Photograph of sample being injected directly into tumor (**B**) IR image of the animal after sample injection (Peak Temp: 29.8 ± 2 °C) (**C**) Photograph of RFA procedure of sample injected liver tumor (**D**) Corresponding IR image after RFA (Peak Temp: 81.3 ± 1.5 °C) (**E**) Photograph of RFA procedure of untreated healthy liver (**F**) IR image of the animal after RFA of untreated healthy liver region (Peak Temp: 51.3 ± 2 °C) (**G**) Graph showing the variation in temperature at the RF treatment point in nCP:Fe treated tumor and control healthy liver without nanoparticle.

To confirm the differential ablative effects in the nanoparticle treated tumor versus untreated liver region, we have done separate experiments where IR thermal imaging was employed to capture the real-time temperature increase during the application of RFA. Two groups (1. Orthotopic liver tumor rats 2. Healthy rat as control), each containing 3 animals were used for this study. In the orthotopic rat liver tumor animals, NP were injected into the tumor region and RFA was applied. Healthy liver without nanoparticle injection was used as control. Figure 8A, B shows visual image and IR thermal image, respectively of sample being injected intratumorally to animals just before the RFA. With the application of RF, 10 W for 1 minute (Fig. 8C), temperature at the RF treatment point raised to 81.3 ± 1.5 °C (starting temp: 29.8 ± 2 °C) in nCP:Fe treated tumor (IR image: Fig. 8D) whereas in the control healthy liver (Fig. 8E), maximum temperature obtained was only 51.3 ± 2 °C (IR image: Fig. 8F) as plotted in Fig. 8G. Effectively, the nCP:Fe nanoparticles caused 119 ± 10% increase in ΔT of tumor region (ΔT_avg_ = 51.5 °C) compared to the control (ΔT_avg_ = 21.5 °C). Thus we demonstrated the capability of nCP:Fe as a theranostic agent for MR assisted RFA of liver tumor.

## Conclusion

In summary, we have developed a novel theranostic biomineral nanoparticle using iron doped calcium phosphate (nCP:Fe) that could provide dual mode (T1-T2) magnetic resonance contrast enhancement together with thermal response suitable for ablation of solid tumors on exposure to clinically approved radiofrequency range and power. Magnetic characterization showed increase in paramagnetic susceptibility with Fe^3+^ doping. Optimum concentration of Fe^3+^ required for dual mode T1-T2 contrast was optimized at 4.1 at % doping with relaxivity values, *r2* = 29.6 mM^−1^s^−1^ and *r1* = 0.75 mM^−1^s^−1^. *In vivo* dual mode MR imaging capability of nanoparticles was demonstrated in rat models. Bio-distribution analysis using whole body T2 imaging and mapping showed maximum liver accumulation by ~1 hour after sample injection followed by complete clearance by ~96 hours. Radiofrequency impedance measurement showed an increase in dielectric loss factor (tan $\delta$) due to doping with Fe^3+^ and the nanoparticles exhibited excellent RF mediated thermal response (100 W, 1 minute) with ΔT = 22 °C in water medium. *In vitro* cell response to RF after nCP:Fe treatment showed 26% increased cell death compared to undoped nCP treated cells. MRI assisted RFA, demonstrated in healthy rat liver, led to enhanced RF mediated necrosis in nCP:Fe treated tissue (necrosis diameter 12 ± 1 mm) compared to PBS control (necrosis diameter 8 ± 1 mm). In orthotopic liver tumor model, nCP:Fe injected tumor region showed 119 ± 10% increase in ΔT (Final temperature: 81.3 ± 1.5 °C) compared to the untreated healthy liver region (Final temperature: 51.3 ± 2 °C). Thus, we demonstrated the synthesis and *in vivo* theranostic application of a novel biomineral nanoparticle, nCP:Fe, for dual-mode MR image assisted radiofrequency ablation of liver tumor. Compared to any other engineered nanoparticles, calcium phosphate, being a major mineral component of our body, possesses excellent translational potential.

## Methods

### Synthesis of nCP:Fe

In a typical reaction procedure, 20 mL of 0.5 M calcium chloride (CaCl_2_, Sigma, USA) was mixed with 20 mL of 0.2 M trisodium citrate (Na_3_C_6_H_5_O_7_, Fisher Scientific, India) and 0.1 M FeCl_3_ (Sigma, USA). Volume of 0.1 M FeCl_3_ added was varied as per the required percentage of doping. 5 mL of 0.3 M diammonium hydrogen phosphate ((NH_4_)_2_HPO_4_, S.D Fine Chemicals, India) mixed with 0.2 mL of 3 N ammonium hydroxide (NH_4_OH, Fisher Scientific, India) was added drop wise to the above mixture of CaCl_2_, Na_3_C_6_H_5_O_7_ and FeCl_3_ under constant stirring to obtain nCP:Fe. The precipitate was washed 4 times in hot distilled water by centrifugation at 8500 rpm for 15 minutes and redispersed in PBS for further studies.

### Characterization of nCP:Fe

XRD of nCP:Fe was analyzed using PANanalytical X Pert-pro system fitted with Cu-Kα source. Particle size analysis was done using transmission electron microscope (Tecnai, G2, FEI) and dynamic light scattering (Nano ZS, ZetasizerNanoseries, Malvern). Zeta potential was measured using Nano ZS, ZetasizerNanoseries, Malvern. Fe^3+^ content within nCP:Fe was analyzed by Inductively Coupled Plasma-Atomic Emission Spectroscopy (ICP-AES Thermo Electron IRIS INTREPID II XSP DUO). 10 mg of nCP:Fe was dissolved in 5% nitric acid for ICP-AES analysis. Magnetic property of nCP:Fe was analyzed in vibration sample magnetometer (ADE EV7 1.5 T). T1 and T2 weighted magnetic contrast imaging was done in 7 T animal MRI station (BrukerBioSpec, USA). 1.25 mg/mL of nCP:Fe with varying concentration of doped Fe^3+^ was dispersed in 1% agar phantoms and MRI was carried out. T2 and T1 weighted MRI parameters were TR = 2500 mS, TE = 33 mS, FA = 180° and TR = 8.5 mS, TE = 2.2 mS, FA = 15° respectively. Relaxivity values, *r1* and *r2*, were calculated from T1 and T2 mapping data for different concentrations of nCP:Fe. Dielectric loss of nCP:Fe was calculated from frequency response impedance measurements in frequency response analyzer (Nova Impedance Spectroscopy). For impedance measurement of nCP:Fe, powder sample was made into pellet and sandwiched between Indium Tin Oxide coated glass slides. To make proper contact silver paste was applied on both sides of pellet and using clips connected to the frequency response analyzer.

### *In vitro* Cell viability and Hemolysis analysis

Hemocompatibility analysis was carried out in human peripheral blood samples collected from healthy volunteers after the approval of the Institutional Human Ethics Committee, Amrita Institute of Medical Sciences and Research Centre, India. Informed consent was obtained from all subjects and all methods for humans were performed in accordance with the WHO guidelines for best practices in phlebotomy. *In vitro* cell viability was tested in PBMC isolated from human blood. PBMC was isolated using density gradient separation using Histopaque 1077, density 1.077 g/mL (Sigma, USA). Cells were redispersed in RPMI complete medium containing 10% FBS, 2 mm L-glutamine and 50 µM β mercaptoethanol. 100 µL of nCP:Fe of varying concentration from 20–500 µg/mL, was taken in 96 well plates. 100 µL of cells at a concentration of 10^5^ cells/mL were seeded into the wells containing the samples. PBS was taken as the negative control and Triton X-100 as the positive control for the experiment. The cells were incubated with nCP:Fe for 48 hours at 37 °C. 100 µL of 10% Alamar blue was added and incubated for 8 hours before the absorbance was measured at 570 and 600 nm. For hemolysis analysis, whole blood was collected in 3.8% trisodium citrate anticoagulant (volume ratio of anticoagulant to blood = 1:9). 450 µL of whole blood was treated with 50 µL of nCP:Fe for 3 hours at 37 °C under mild mixing. PBS and 1% Triton × 100 was taken as the negative and positive control respectively. Blood was then centrifuged at 4000 rpm for 15 minutes to collect plasma. Absorbance of plasma diluted with 0.01% sodium carbonate solution was measured spectrophotometrically (UV-1700, Shimadzu) at 380, 415 and 450 nm. Amount of plasma hemoglobin was calculated as in Equation 1.

1 $$Amount\,of\,plasma\,hemoglobin\,(mg/dL)=\frac{2\times {A}_{415}-({A}_{380}+{A}_{450})\times 1000\times Dilution\,factor}{(E\times 1.655)}$$

where A_415_, A_380_, A_450_ are the absorbance values at 415, 380 and 450 nm. A_415_ is the soret band based absorption of hemoglobin. A_380_ and A_450_ are correction factors applied for uroporphyrin absorption. E is molar absorptivity value of oxyhemoglobin at 415 nm (E = 79.46). 1.655 is the correction factor applied due to the turbidity of plasma sample. % Hemolysis was calculated as in Equation 2.

2 $$ \% \,Hemolysis=\frac{Plasma\,Hb\,value\,of\,sample}{Total\,Hb\,value\,of\,blood}\,\times 100$$

where Hb is hemoglobin. For SEM imaging, RBC after particle treatment was incubated with 2.5% glutaraldehyde for 30 minutes. The cells were then washed twice in PBS before SEM imaging was carried out.

### ROS generation analysis

DCFH-DA assay was used to measure the intracellular ROS after treatment of nCP:Fe with N1S1 hepatoma cells. Cells were seeded at a density of 2 × 10^5^ cells per well in 6 well plates. nCP:Fe at a concentration of 250 µg/mL was added to cells. PBS and 30 µM H_2_O_2_ served as negative and positive controls respectively. The cells were incubated for 12 hours (4 hours for positive control) and washed with PBS. 20 µM of DCFH-DA in buffer was added to cells and incubated for 30 minutes at 37 °C. The cells are taken for flow cytometry (BDFACSAria II), intensity of fluorescence was detected with an excitation filter of 488 nm and band-pass emission filter of 530 ± 15 nm.

### *In vivo* MRI and Bio-distribution analysis

All animal procedures were approved by the Institutional Animal Ethics Committee, Amrita Institute of Medical Sciences and Research Centre and the methods were done in accordance with guidelines of CPCSEA (Committee for the Purpose of Control and Supervision of Experiments on Animals, Govt. of India) guidelines. *In vivo* MRI was carried out in 7 T animal MRI station (BrukerBioSpec, USA). 10 mg/kg of nCP:Fe was injected intravenously into wistar rat. The animal was anesthetized in an induction chamber of isoflurane - oxygen mixture (5% isoflurane). Rat was placed on the animal bed with a mask for providing maintenance dose of the anesthesia (2% isoflurane). A respiration pad and temperature probe with circulating warm water was used for continuous monitoring of respiration rate and body temperature. For T_1_ and T2 weighted MRI, a T_1_ FLASH imaging sequence with TR = 64 mS, TE = 2.2 mS, FA = 15°and Turbo-RARE T2 imaging sequence with TR = 2500 mS, TE = 33 mS, FA = 180° was used respectively. For calculating T2 value of liver, T2 mapping sequence was carried out in axial liver sections. 3 different ROI was selected and the average T2 value was estimated. Biodistribution analysis was also carried out using the same parameters over a period of 96 hours. T1 and T2 weighted MRI of nCP:Fe injected intratumorally to subcutaneous tumor was also done using the above mentioned parameters. Organs were collected after euthanasia of animals by an overdose of the anesthetic (Xylazine: Ketamine = 1:4). After washing each organ with PBS, it was weighed and homogenized. The homogenized tissue was dissolved in Tissue solvable (PerkinElmer) over a period of 24 hours and made up to 50 mL with 5% nitric acid for ICP-AES analysis to estimate Fe^3+^ content.

### Prussian blue staining of RAW 264.7 macrophages

Cells were seeded to 24 well culture plates at a seeding density of 10,000 cells/ well and incubated overnight for cell attachment. The medium was then replaced with opti-MEM medium containing 100 ug/mL of nCP:Fe and incubated for 12 hours. Medium was removed and cells were cultured in DMEM complete medium for 6 hours. Cells were then washed with PBS and fixed using 4% paraformaldehyde (Merck Co, India). Cells were then washed and treated with a mixture of 5% potassium ferrocyanide (Sigma-Aldrich Co, USA) and 5% HCl (VeTEC, India) for 10 minutes. Cells were washed and stained with nuclear fast red (Merck- Millipore, USA) for 5 min. Dehydration followed by mounting of coverslips was done. The cells were examined under optical microscope (Olympus, BX5).

### *In vitro* RF response of Nanoparticles

RF response of the nanoparticles was measured in a custom made non-invasive 13.5 MHz RF instrument. Different concentrations of nCP:Fe varying from 10–500 µg/mL was taken in a small glass petridish and 100 W RF power was applied for 1 minute. The temperature of the solution was measured before and after RF irradiation. RF response of nanoparticle treated N1-S1 hepatoma cells was also tested using the same non-invasive RF instrument. N1-S1 cells were seeded in 24 well plates at a seeding density of 2.5 × 10^4^ cells/well. Different concentrations of nCP:Fe (50–500 µg/mL) was added to the wells. After incubation with the nanoparticles for 4 hours, the cells were irradiated with 100 W RF power for 5 minutes. 4 hours after RF treatment, media was changed. After 48 hours, cell viability analysis was carried out using Alamar blue assay. For estimation of RF response in tissue phantom, we chose goat liver tissue and invasive RF procedure was carried out using a cool tip RF ablation system (Cool-tip^TM^ RF ablation system, E series Covidien). The experiment was carried out in goat liver collected from 3 different animals, divided to two groups (i) PBS control group and (ii) nCP:Fe group. 10 mg/kg of nCP:Fe was injected to goat liver at a depth of 1 cm. PBS was injected to control tissue. The RF probe was inserted (near to the point of sample injection) to a depth of 1 cm within the tissue and a power of 10 W was applied for 1 minute. After RF ablation, infrared (IR) image of the tissue was taken using IR imager (Fluke Ti200 Infrared camera) and diameter of the area of coagulation necrosis was measured.

### *In vivo* Radiofrequency Ablation in Healthy Liver and Orthotopic Liver Tumor Model

Healthy adult wistar rats were divided into two groups (PBS control group and nCP:Fe group) containing 3 animals each. 10 mg/kg of nCP:Fe/PBS was injected intravenously to the rats. MRI was carried out before and after sample injection. Animals were then given an overdose of anesthesia. The abdomen region was opened and RF probe was inserted into the liver up to 1 cm depth. 10 W RF power was applied for a period of 1 minute using clinically used RFA system (Cool-tip^TM^ RF ablation system, E series Covidien). MRI of axial liver sections was carried out using TurboRARE T2 imaging sequence (TR = 2500 mS, TE = 33 mS, FA = 180°). The animals were then euthanized and liver was collected. ~2 mm thick liver tissue was cut and live tissue staining was carried out using 2,3,5 Triphenyltetrazolium chloride (TTC) stain. The liver sections were incubated with 1% TTC stain at a temperature of 37 °C for 30 minutes. Hematoxylin and Eosin staining was done to observe the extent of necrosis in tissue sections.

For development of orthotopic rat liver tumor models, 4–6 weeks old Sprague Dawley rats were injected with 1 × 10^6^ N1-S1 rat hepatomacells (N1S1) under the capsule of left hepatic lobe. 10 days after injection tumor reached a size of ~1.5 cm^3^. 300 µL of 10 mg/mL sample was injected intratumorally to half of the tumor lobe. MRI was done to assess the particle distribution. RFA of the tumor was carried out at 10 W for 1 minute using clinically used RFA system (Cool-tip^TM^ RF ablation system, E series Covidien). TTC staining of the liver was done to compare the viability of injected and control tumor region. The experiment was repeated in 3 orthotopic liver tumor rat models to obtain the average variation in necrosis area. Next, comparison of the increase in temperature of sample injected tumor region *vs*. healthy liver was carried out. Two groups (1. Orthotopic liver tumor rats 2. Healthy rat as control), each containing 3 animals were used for this study to obtain the average variation in tissue temperature. RFA (using Cool-tip^TM^ RF ablation system, E series Covidien) was done at 10 W for 1 minute. IR image (using Fluke Ti200 Infrared camera) was taken soon after RFA to compare the temperature increase in sample injected tumor vs control liver region.

## Electronic supplementary material

Supplementary Information
