# Supplementary material for: Biomineral Nano-Theranostic agent for Magnetic Resonance Image Guided, Augmented Radiofrequency Ablation of Liver Tumor
Source: Sci Rep. 2017 Nov 3;7:14481. doi: 10.1038/s41598-017-14976-8 (PMC5670219; doi:10.1038/s41598-017-14976-8)
Supplement: Supplementary file 1 — Supplementary Information [file 41598_2017_14976_MOESM1_ESM.pdf]

Supporting Information

Biomineral Nano-Theranostic agent for Magnetic Resonance Image Guided, Augmented Radiofrequency Ablation of Liver Tumor

Anusha Ashokan, Vijay Harish Somasundaram, Genekehal Siddaramana Gowd, Ida M Anna,

Giridharan L Malarvizhi, Badrinathan Sridharan, Rupal B Jobanputra, Reshmi Peethambaran,

A.K.K. Unni, Shantikumar Nair, Manzoor Koyakutty

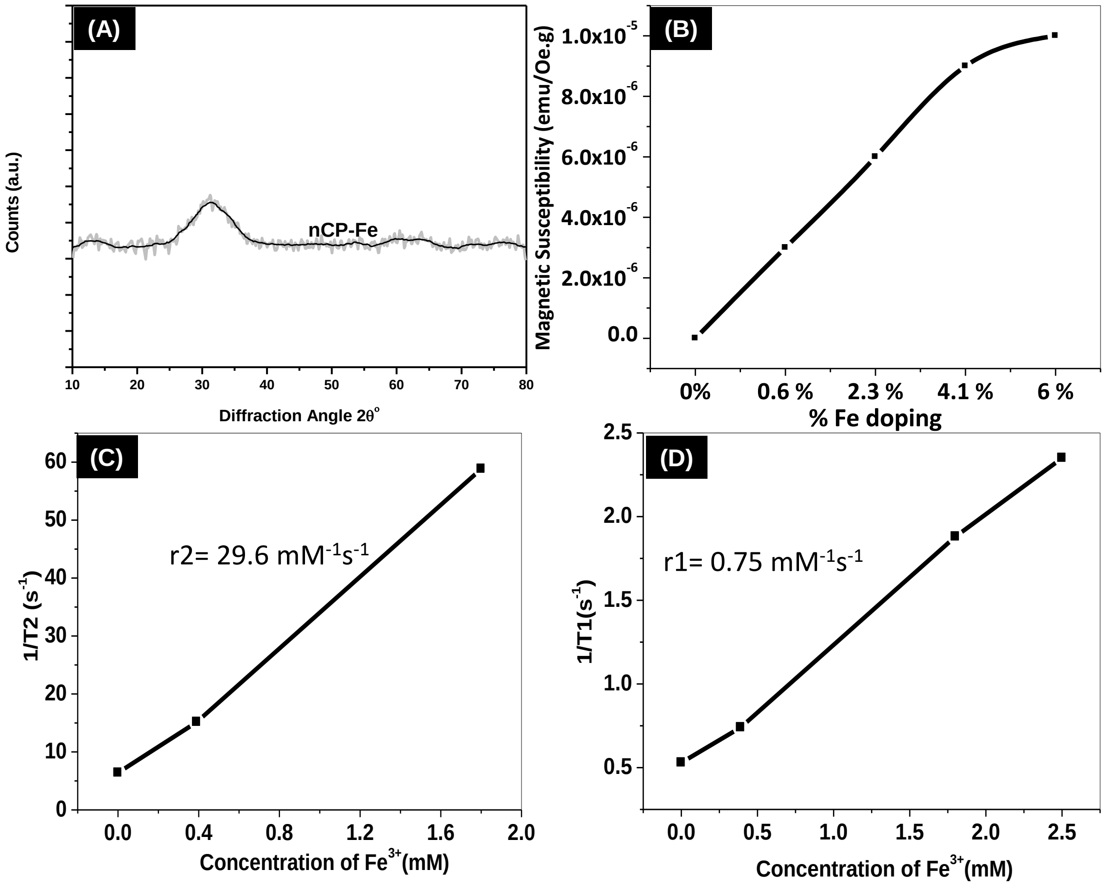

Figure S1. (A) XRD of nCP:Fe (B) Magnetic susceptibility data of different batches of nCP:Fe doped with varying concentration of Fe^{3+} (B) r2 and (C) r1 relaxivity plot of 4.1 at % Fe^{3+} doped nCP:Fe

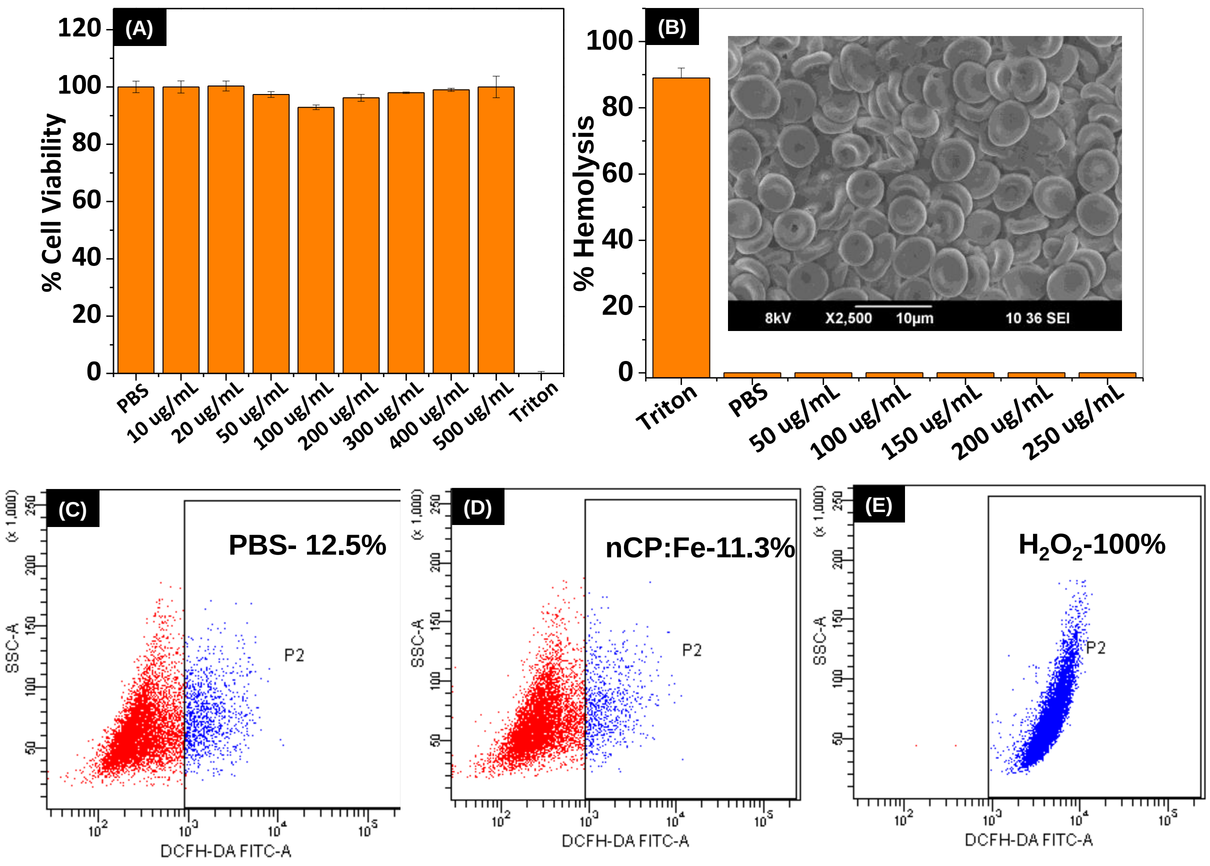

Figure S2. *In vitro* toxicity analysis of nCP:Fe. (A) Viability of PBMC after incubation with nCP:Fe for 48 hours (B) Hemolysis data after incubation of whole blood with nCP:Fe for 3 hours. Inset: SEM showing intact RBC membrane after nCP:Fe treatment. Flow cytometry based ROS analysis after treatment of N1S1 cells with (C) PBS (D) nCP:Fe and (E) H_2O_2.

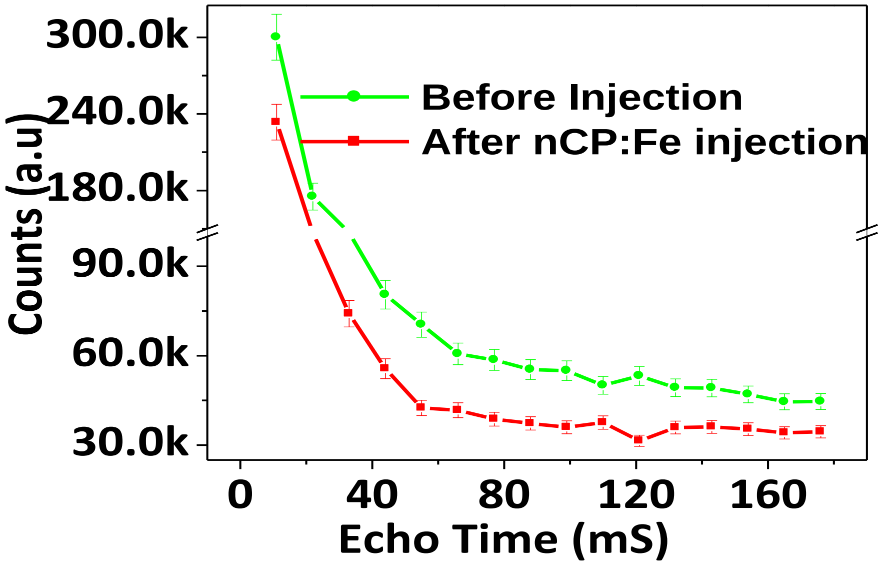

Figure S3. T2 mapping data averaging 3 ROI selected in the axial sections (as shown in Figure 2 C&D) before and 1 hour after nCP:Fe injection.

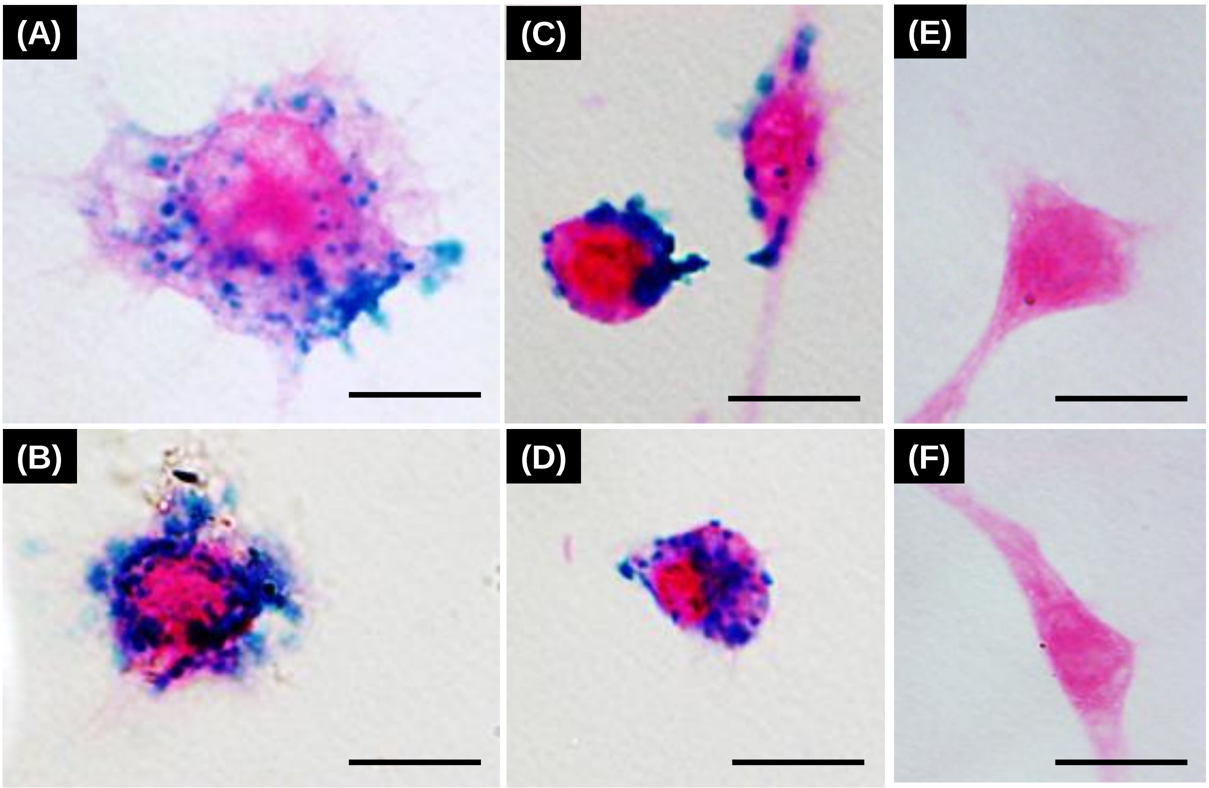

Figure S4: Prussian blue stained RAW 264.7 macrophages (A-D) after treatment with nCP:Fe for 12 hours (E,F) control cells without nanoparticle treatment. Bar represents 10 µm.

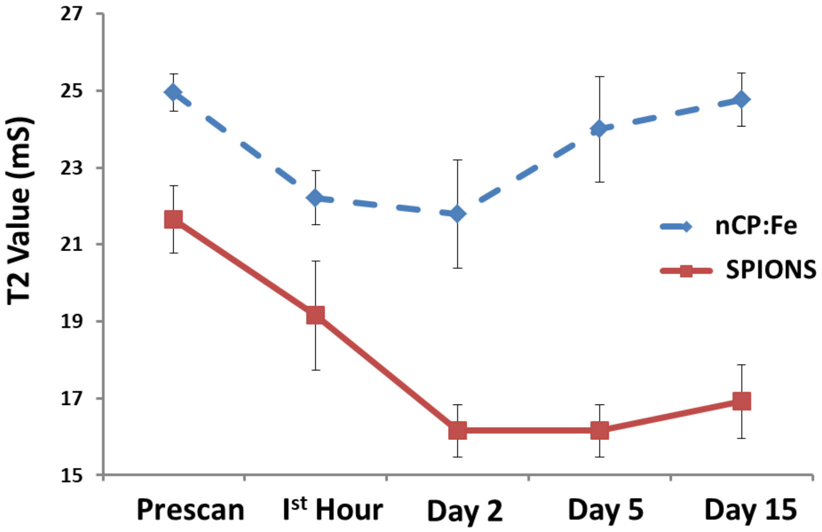

Figure S5: Comparison of T2 values in liver after intravenous injection of nCP:Fe vs SPIONS.

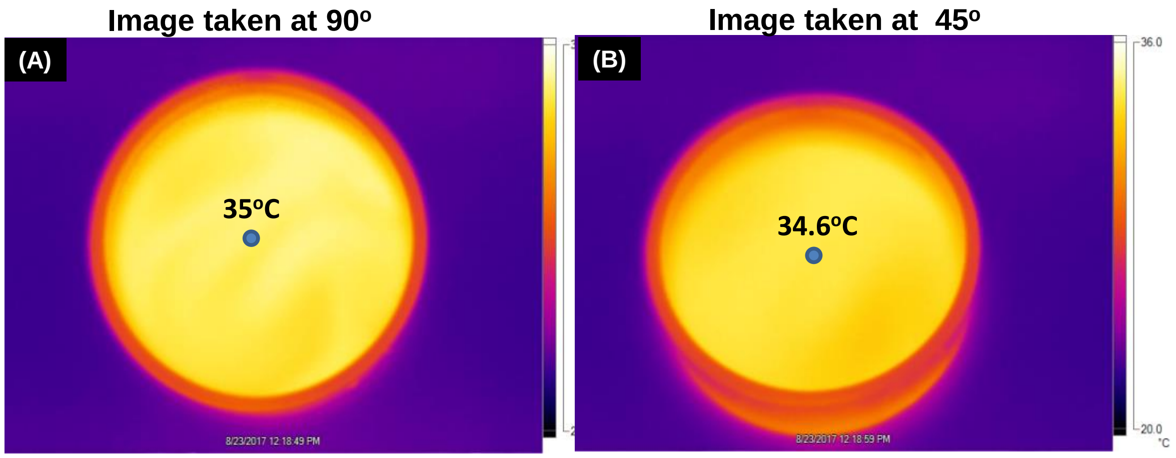

Figure S6: IR image and corresponding temperature of distilled water heated to 35°C.

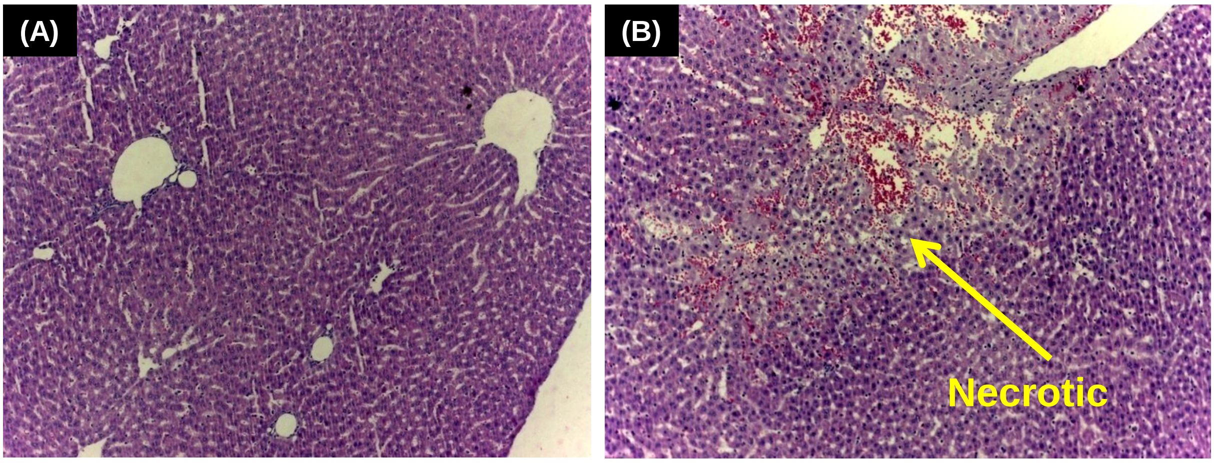

Figure S7. Histology sections of liver from animal injected with nCP:Fe (A) region not treated with RF (B) region exposed to RF showing necrosis
